# Tracking Diet Preferences of Bats Using Stable Isotope and Fatty Acid Signatures of Faeces

**DOI:** 10.1371/journal.pone.0083452

**Published:** 2013-12-23

**Authors:** Monika My-Y Lam, Dominik Martin-Creuzburg, Karl-Otto Rothhaupt, Kamran Safi, Elizabeth Yohannes, Ioanna Salvarina

**Affiliations:** 1 Limnological Institute, University of Konstanz, Konstanz, Germany; 2 Department of Migration and Immuno-ecology, Max Planck Institute for Ornithology, Radolfzell, Germany; 3 Department of Biology, University of Konstanz, Konstanz, Germany; University of Regina, Canada

## Abstract

Stable isotope and fatty acid signatures of biomaterials can provide important information about the dietary niche of animals. Stable isotope and fatty acid signatures differ between aquatic and terrestrial food webs, and therefore can be used to assess the aquatic and terrestrial contributions to the diets of species. We studied faecal samples of three co-occurring bat species with known differences in feeding preferences. The aim was to assess whether stable isotope and fatty acid signatures of faeces can be used to determine feeding preferences. We used bat faeces because they can be easily and non-invasively collected. We hypothesised that faeces stable isotope and fatty acid signatures will reveal the terrestrial, aquatic and mixed feeding niches of *Myotis myotis, M. daubentonii*, and *M. mystacinus*, respectively. As predicted, the faeces of *M. myotis* were characterized by higher *δ* ^13^C values and higher concentrations of linoleic acid and total ω6 polyunsaturated fatty acids (PUFAs), which are typically higher in terrestrial food webs. The faeces of *M. daubentonii* had higher *δ* ^15^Ν values and higher concentrations of docosahexaenoic acid and total ω3 PUFAs, characteristic features of aquatic systems. *Myotis mystacinus* faeces had intermediate *δ* ^15^Ν values and concentrations of both types of fatty acids. Our results show that analysing stable isotope and/or fatty acid signatures of faeces provides a promising, non-invasive tool to study the feeding ecology of bats and to assess aquatic-terrestrial interactions.

## Introduction

Biochemical tracers, such as stable isotopes and fatty acids, can provide useful information on feeding habits and ecological niches of animals [1]. They can help to understand food web complexity, the coexistence of species, and to elucidate mechanisms maintaining species diversity. In addition, identifying an animal’s preferred habitats is crucial for establishing conservation management plans. Since these tracers are different between different habitats, they can be applied to explore species’ flexibility in changing feeding habitats, for example from terrestrial to aquatic. In many areas, aquatic food sources are limited or suffer due to anthropogenic changes and pollution. Biochemical tracers can be also applied to investigate species’ dependence on aquatic resources, and to assess the degree of aquatic subsidies into terrestrial systems and vice versa, which is important for the understanding of the ecosystem functioning.

Bats, among other terrestrial organisms, can depend partially or entirely on aquatic prey for their nutrition. We used collections of bat faeces to investigate whether the stable isotope and fatty acid tracers could discriminate between aquatic and terrestrial feeding preferences in bats. Stable isotope and fatty acid signatures have been analysed in samples derived from the capture of animals to acquire blood, muscle, skin, breath, adipose tissue, liver or the entire carcass [2]–[5]. Collecting these samples is not only laborious, particularly for nocturnal animals, but is also invasive and it should be minimised.

In contrast, collecting faeces is easy and cost-efficient and is usually completely non-invasive when collected, for example, from below bat roosting sites. Faeces have rarely been subjected to stable isotope analysis (but see [6]–[8]) and to the best of our knowledge, never to fatty acid analysis for samples from bats or other small mammals. Visual identification of prey remains in bat faeces is common [9], [10], but time consuming and requiring expertise in insect identification. Further, it cannot always be used to discriminate the origin of prey (e.g. aquatic or terrestrial). Molecular techniques, which also have been applied to faeces to study the composition of prey species [11], are more complete but at the cost of being expensive.

Stable isotopes can be used to trace the sources of organic matter to terrestrial or aquatic systems [12], as different food webs exhibit different isotopic signatures. Aquatic and terrestrial isotope signatures vary regionally, but within the same region, freshwater biota often have higher stable nitrogen isotope (*δ* ^15^N) and lower stable carbon isotope (*δ* ^13^C) signatures than terrestrial biota of comparable trophic levels [13]–[15]. Isotopic signatures also differ between freshwater and marine invertebrates [16], [17]. Stable sulphur isotope values (*δ* ^34^S) are generally higher in freshwater compared to terrestrial ecosystems [18], but do not always discriminate freshwater from terrestrial consumers [19].

The content and composition of polyunsaturated fatty acids (PUFAs) in organic matter also typically differs between aquatic and terrestrial systems as well as between marine and freshwater sources. Marine invertebrates usually contain higher proportions of ω3 (omega-3, n-3) PUFAs compared to freshwater invertebrates which often have higher proportions of ω6 (omega-6, n-6) PUFAs [20]. Marine food webs have 5 to 20 fold higher concentrations of ω3 PUFAs than ω6 PUFAs [21]. In terrestrial food webs, ω6 PUFAs are more abundant than ω3 PUFAs [22], [23]. Thus, the concentrations of ω3 and ω6 PUFAs and the ratio of ω3/ω6 can be informative for assessing the relative contribution of aquatic and terrestrial food in an organism’s diet. Mammals feeding on aquatic prey have tenfold higher concentration of docosahexaenoic acid (DHA, 22:6n-3, ω3 PUFA) in peritoneal adipose tissue than species feeding on terrestrial diets (European otter, *Lutra lutra* compared to stone marten, *Martes foina* and European wild cat, *Felis sylvestris*) [24]. In contrast, linoleic acid (LIN, 18:2n-6, ω6 PUFA) is more concentrated in mammals feeding on terrestrial diets [24]. The ratio DHA/LIN has been proposed as a proxy for following changes in terms of aquatic and terrestrial contributions in the diets of carnivorous mammals [24].

The aim of our study was to investigate the suitability of stable isotope and fatty acid signatures from faecal samples to detect aquatic versus terrestrial prey items of different bat species. We expected that differences in these tracers between aquatic and terrestrial prey organisms would be reflected in the stable isotope and fatty acid signatures of faeces from bats feeding on aquatic or terrestrial prey. Earlier, in a diet-switching experiment, we confirmed that bat faecal stable isotopes reflect the signature of the most recent food with a turnover rate of 2–3 hours [25]. We analysed the faeces of three bat species from the genus *Myotis* inhabiting the same region but with different feeding preferences in terms of aquatic and terrestrial prey. *Myotis myotis* (greater-mouse eared bat, Borkhausen 1779) has been reported to prey on terrestrial arthropods, especially Carabidae but also on Grillidae, Arachnida, and larvae of Lepidoptera in open areas, fresh cut meadows or forests [26]. *Myotis daubentonii* (Daubenton’s bat, Kuhl 1817) is known to hunt over still waters or slow moving rivers and mainly preys on Chironomidae emerging from the water [27]–[29]. *Myotis mystacinus* (Whiskered bat, Kuhl 1819), appears to be more flexible in foraging behaviour, is known to hunt in parklands, woodlands and over running water [30], where it mostly feeds on Diptera (Tibulidae, Chironomidae, Anisopodidae), but these bats have also been reported to consume Arachnida and Lepidoptera [27], [31].

We predicted that faeces of *M. myotis*, the terrestrial feeder, would display terrestrial signatures, with higher proportions of LIN and total ω6 PUFAs, higher *δ* ^13^C and lower *δ* ^15^Ν values. *Myotis daubentonii*, the aquatic feeder, was expected to have an aquatic signature, i.e. higher proportions of DHA and total ω3 PUFAs, lower *δ* ^13^C and higher *δ* ^15^N values. For *M. mystacinus* which feeds both on aquatic and terrestrial insects, we expected an intermediate signature. Finding an aquatic or terrestrial signature for individual *M. mystacinus* faecal pellets would not be surprising, given that they might have been produced by individuals that had consumed more of one prey type than the other.

## Materials and Methods

### Ethic statement

Sampling was conducted in collaboration with bat conservation organizations active in Konstanz and Kreuzlingen (‘Arbeitsgemeinschaft Fledermausschutz BW e.V.’ and ‘Fledermausschutz Thurgau’, respectively). The species we studied are listed as ‘of least concern’ according to the IUCN red list [32]. All samples were collected at privately owned buildings after asking for permission from the owner or manager. No special permissions were required as the animals were not disturbed.

### Sample collection

**Figure 1:**
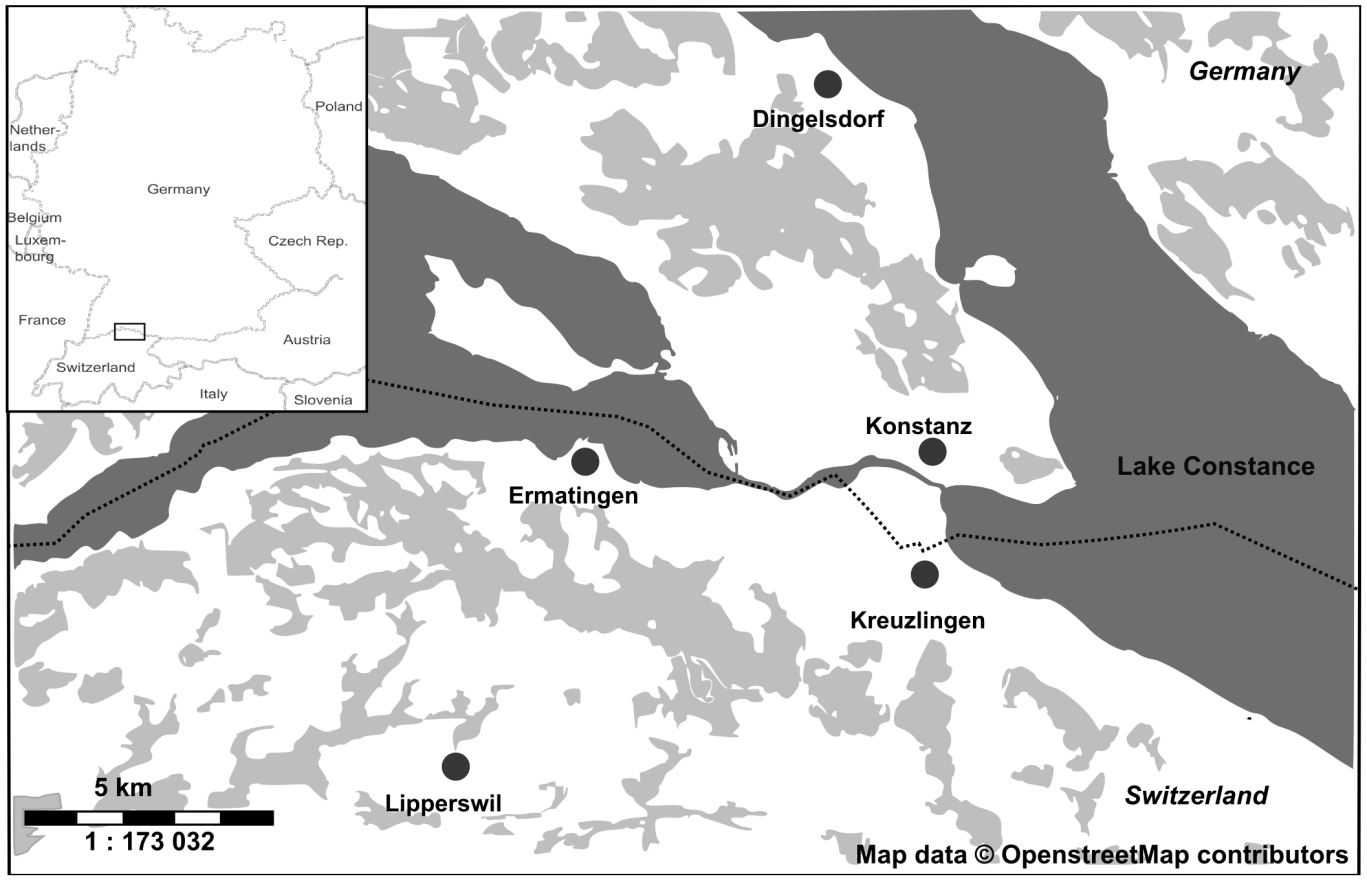
Map of sampling locations. Map of faecal sampling locations (solid circles) for *Myotis myotis* (Ermatingen and Lipperswil), *M. mystacinus* (Dingelsdorf) and *M. daubentonii* (Kreuzlingen). In the top left the broad sampling area is marked in a box (modified from © OpenStreetMap contributors).

Faecal samples were collected in Switzerland and Germany in the vicinity of Lake Constance (Figure 1). To collect fresh faeces from roosts, we placed a plastic sheet on the floor, underneath the bats, the day before collection. In the end of April on the same day, we collected faeces of *M. myotis* in attics of churches located in Ermatingen and in Lipperswil (both in Switzerland), which are approximately 0.5 km and 6.5 km from Lake Constance, respectively. From Lipperswil we also collected samples from May to June 2011. Faeces of *M. daubentonii* were collected, in May and June 2011, from a hospital attic in Kreuzlingen (Switzerland), approximately 1 km from Lake Constance. Faeces of *M. mystacinus* were collected in May 2011, from behind a shutter on a house in Dingelsdorf, Konstanz (Germany), approximately 0.5 km from Lake Constance. We transported samples to the laboratory and stored them at –80°C until further processing.

We analysed 6 faecal samples for stable isotopes and another 6 for fatty acids per sampling date for each species. The pellets were chosen by selecting the first pellets that forceps touched in the sample container. A total of 71 samples were analysed for stable isotopes and another 71 for fatty acids (in each case: *M. myotis*: n = 29, *M. mystacinus*: n = 24 and *M. daubentonii*: n = 18).

### Stable isotope analysis

Faeces were oven dried at 50°C, ground and 1.5±0.001 mg was weighed in tin capsules on a microbalance (Mettler Toledo Excellence Plus XP6). A sample consisted of one faecal pellet, except in a few cases where two pellets from *M. mystacinus* had to be used due to the small faeces of this species. Stable isotope analyses for nitrogen (*δ* ^15^N), carbon (*δ* ^13^C) and sulphur (*δ* ^34^S), were conducted on the same sample, combusted in a Micro cube (Elementar, Germany) elemental analyser (Limnological Institute, University of Konstanz, Germany). The resulting N_2,_ CO_2_ and SO_2_ were separated by gas chromatography and admitted into the inlet of a Micromass (Isoprime, UK) Isoprime isotope ratio mass spectrometer (IRMS) for determination of ^15^N/^14^N, ^13^C/^12^C and ^34^S/^32^S, respectively. Replicate standards of sulphanilamide (Isoprime internal standards) and casein (source: Elementar Analysensysteme GmbH, Germany) were used as laboratory standards for every 8 unknown samples in sequence. The measurements are reported in *δ*-notation (*δ* ^15^N, *δ* ^13^C, *δ* ^34^S, respectively) in parts per thousand deviations (‰), where *δ*  =  1000 x (Rsample/Rstandard) –1 ‰, relative to atmospheric N_2_ for nitrogen, to the Pee Dee Belemnite (PDB) for carbon, and sulphanilamide calibrated and traceable to NBS-127 (barium sulphate) for sulphur. R =  heavy/light isotopes: ^15^N/^14^N, ^13^C/^12^C,^ 34^S/^32^S. Internal laboratory standards indicate that our measurement errors (SD) were ± 0.15‰, 0.05‰ and 0.05‰ for *δ* ^15^N, *δ* ^13^C, and *δ* ^34^S, respectively.

### Fatty acid analysis

The lipids were extracted twice from approximately 10 mg of freeze-dried faecal samples (usually 2–4 pellets) with dichloromethane/methanol (2∶1 v/v). The pooled extracts were evaporated to dryness with nitrogen. Fatty acids were transesterified with 3 mol L^−1^ methanolic HCl (60°C, 20 min). Fatty acid methyl esters (FAMEs) were extracted three times with 2 ml iso-hexane. The combined extracts were evaporated to dryness with nitrogen and resuspended in 10 µl iso-hexane. FAMEs were analyzed by gas chromatography (GC) on an HP6890. The GC was equipped with a flame ionization detector and a DB-225 (J & W Scientific) capillary column. Details of GC configurations are given elsewhere [33]. FAMEs were identified by comparing retention times with that of reference substances (Supelco FAME standard, complemented by 18:1n-9, 18:4n-3, 20:1n-7) and quantified by comparison to internal standards (17:0 ME and 23:0 ME) of known concentrations using multipoint calibration curves determined previously with lipid standards. The identification of fatty acids was verified by analyzing mass spectra recorded in selected samples using a gas chromatograph-mass spectrometer (GC-MS; Agilent Technologies, 5975C inert MSD) as described before [33].

For the evaluation of fatty acids, we summed all ω3 and ω6 PUFAs and also calculated the ω3/ω6 ratio. We also considered single ω3 and ω6 PUFAs, i.e., DHA and LIN. The fatty acid data were evaluated and represented as percentages of total fatty acids present in a sample (%TFA, Total Fatty Acid).

### Statistical analyses

We checked if the stable isotope (*δ* ^15^N, *δ* ^13^C, *δ* ^34^S) and fatty acid (ω3, ω6, DHA, LIN) data deviated significantly from a normal distribution (Shapiro-Wilk test, p>0.05). For Gaussian distributed data or data that could be transformed into a normal distribution we applied parametric tests (ANOVA), else we applied non-parametric tests (Kruskal-Wallis). To assess whether the parameters (i.e., stable isotopes, ω3, ω6, ω3/ω6, DHA, LIN and DHA/LIN values) were different between the two populations of *M. myotis* at the near versus far from the lake locations (Ermatingen and Lipperswil, respectively) we compared the values of all parameters from the two sites (n = 6 per site) using t-tests. Since there was no significant differences (p>0.05) in any parameters, except *δ* ^34^S, the samples were pooled for further analysis.

We used analysis of variance (ANOVA) or the non-parametric Kruskal-Wallis test to investigate differences between the three species (for each parameter separately) and subsequent post-hoc tests (Tukey’s HSD). The tests for the fatty acids were conducted on arcsin-transformed values of the proportions. The ANOVAs were done on i) a dataset with samples summed over all the dates for each species and ii) a dataset with only the samples collected during the same 2 weeks (*M. myotis*: 25^th^ May, *M. mystacinus*: 18^th^ May and 31^st^ May, *M. daubentonii*: 17^th^ May). We chose to compare only these samples, as they were collected on days close to each other and because we could not collect samples on the same day for logistic reasons.

We used general linear models (GLMs) with species and sampling date as factorial covariates to determine whether any explained the variation in each parameter. To investigate the ability to assign the samples to the correct species based on the stable isotope or fatty acid values we performed a linear discriminant function analysis separately for the stable isotope (*δ* ^15^N, *δ* ^13^C, *δ* ^34^S) and fatty acid data (ω3, ω6, ω3/ω6). All statistical analyses were performed using R version 2.15.2 [34]. The package ‘sciplot’ [35] was used for plotting means and standard errors, the package ‘pgirmess’ for the multiple comparisons after the Kruskal-Wallis tests and the package ‘MASS’ [36] was used for the linear discriminant analysis.

## Results

### Stable isotopes

**Figure 2:**
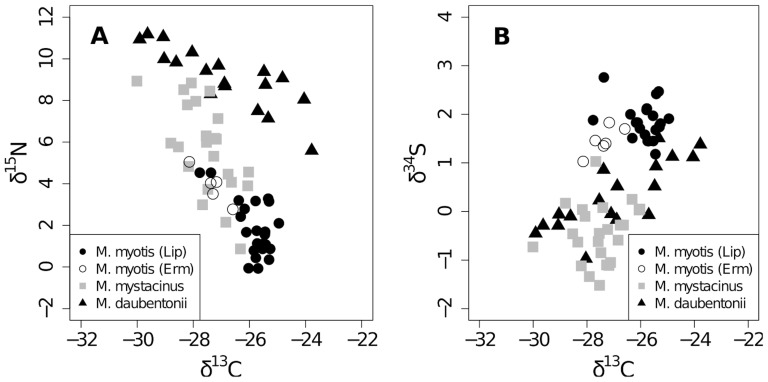
Stable isotope values A. *δ* ^15^N versus *δ* ^13^C and B. *δ* ^34^S versus *δ* ^13^C. Plot of stable isotope values A. *δ* ^15^N versus *δ* ^13^C and B. *δ* ^34^S versus *δ* ^13^C for *Myotis myotis*, *M. mystacinus* and *M. daubentonii*. Values indicate that each species occupied a different isotopic niche. The population of *M. myotis* (Ermatingen) (open circles), which roosted closer to water, occupied the same niche as the population farther away (full circles).

The values of *δ* ^15^N were different between all three species (Kruskal-Wallis, df = 2, X^2^ = 48.31, p<0.001) (Table 1). *Myotis daubentonii* (mean±se: 9.10±1.44‰) faeces were more enriched in *δ* ^15^N than *M. myotis* (mean±se: 1.87±1.32‰), while *M. mystacinus* had intermediate values (mean±se: 5.69±1.99‰) (Figure 2A). The differences in *δ* ^13^C were less pronounced (Figure 2A, Figure 2B). *Myotis myotis* and *M. mystacinus* differed in their *δ* ^13^C values (ANOVA, post-hoc test, F_2,68_ = 8.37, p<0.001), while the *δ* ^13^C values for *M. daubentonii* did not differ from *M. myotis* (ANOVA, post-hoc test, F_2,68_ = 8.37, p = 0.097), nor from *M. mystacinus* (ANOVA, post-hoc test, F_2,68_ = 8.37, p = 0.262) (Table 1). The values of *δ* ^34^S (Figure 2B) were different between *M. myotis* and the other two species (Kruskal-Wallis, df = 2, X^2^ =  54.03, p<0.001) (Table 1).

**Table 1:** Results of the ANOVAs and post-hoc tests or Kruskal-Wallis for the comparison of the stable isotope values between the species, using i) all the samples and ii) the samples collected in the middle of May only.

	all samples			samples only from mid May		
	*δ* ^15^N	*δ* ^13^C	*δ* ^34^S	*δ* ^15^N	*δ* ^13^C	*δ* ^34^S
p value	**<0.001**	**<0.001**	**<0.001**	**<0.001**	**<0.001**	**<0.001**
statistics	df = 2, X^2^ = 48.31	F_2,68_ = 8.37	df = 2, X^2^ = 54.03	F_2,21_ = 38.06	F_2,21_ = 49.10	df = 2, X^2^ = 13.10
species with difference	all	mys-myo	myo-dau/ myo-mys	all	all	myo-dau/ myo-mys
test	Kruskal-Wallis	ANOVA	Kruskal-Wallis	ANOVA	ANOVA	Kruskal-Wallis

myo: *Myotis myotis*, mys: *M. mystacinus*, dau: *M. daubentonii.* The statistically significant values are indicated in bold.

**Figure 3:**
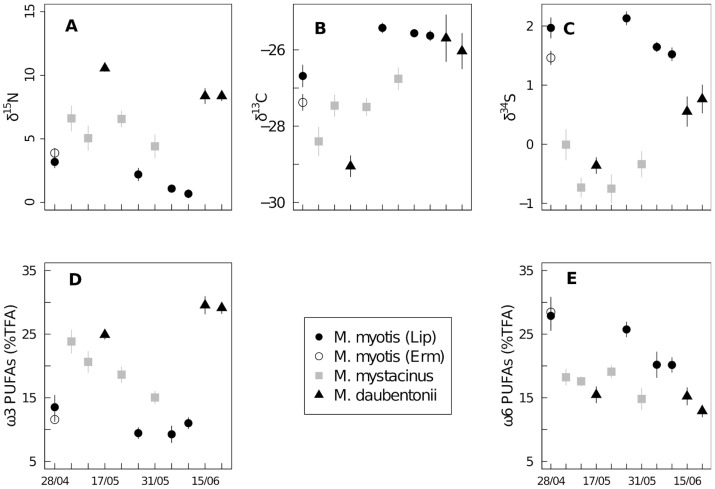
Temporal patterns of stable isotope and PUFA values per species. Temporal patterns (means ± SE) in A. *δ* ^15^N, B. *δ* ^13^C, C. *δ* ^34^S, D. ω3 PUFAs and E. ω6 PUFAs values for *Myotis myotis*, *M. daubentonii* and *M. mystacinus*. Lip: Lipperswil, Erm: Ermatingen.

A strong temporal change occured in the stable isotope values in the faeces of *M. daubentonii* (ANOVAs, for all isotopic elements: p<0.005), with an increasing trend in *δ* ^13^C and *δ* ^34^S (Figure 3, Table 2). The temporal differences in the isotopic values of the faeces of *M. myotis* were more pronounced for *δ* ^13^C (ANOVA, F_3,25_ = 21.03, p<0.001) and for *δ* ^15^N (ANOVA, F_3,24_ = 13.85, p<0.001) showing a decreasing trend (Figure 3, Table 2). A weak temporal change (ANOVA, F_3,19_ = 5.03, p = 0.009) was noted in *δ* ^13^C values in the faeces of *M. mystacinus* (Figure 3, Table 2). When we compared *δ* ^13^C, *δ* ^15^N and *δ* ^34^S between the species using only the samples collected during the same period (middle of May), all values for all species pairs were different (ANOVAs for *δ* ^15^N and *δ* ^13^C, Kruskal-Wallis for *δ* ^34^S, p<0.001), except *δ* ^34^S values between *M. mystacinus and M. daubentonii* (Table 1). The GLMs showed that variation in the *δ* ^13^C and *δ* ^15^N values was explained both by species identity (for *δ* ^13^C: *M. myotis* and *M. daubentonii*: p<0.001 and for *δ* ^15^N: for all species p<0.001) and date (for both isotopes p<0.001) (Table 3). The variation in *δ* ^34^S was explained by species (*M. myotis*: p<0.001 and *M. mystacinus*: p<0.001) while date was not significant (p = 0.094) (Table 3).

**Table 2:** Results of the ANOVAs and Kruskal-Wallis tests for the comparison of the stable isotope and fatty acid values between the different sampling dates for each species.

		*M. myotis*	*M. mystacinus*	*M. daubentonii*
*δ* ^15^N	p value	**<0.001**	0.216	**0.003**
	statistics	F_3,24_ = 13.85	F_3,19_ = 1.63	F_2,15_ = 8.61
	test	ANOVA	ANOVA	ANOVA
*δ* ^13^C	p value	**<0.001**	**0.009**	**<0.001**
	df/F	F_3,25_ = 21.03	F_3,20_ = 5.03	F_2,15_ = 15.34
	test	ANOVA	ANOVA	ANOVA
*δ* ^34^S	p value	**0.030**	0.083	**0.005**
	statistics	F_3,25 = _3.51	F_3,20_ = 2.57	F_2,15_ = 7.75
	test	ANOVA	ANOVA	ANOVA
ω3	p value	0.090	**0.004**	**0.011**
	statistics	F_3,25_ = 2.42	F_3,20_ = 6.26	F_2,15_ = 6.13
	test	ANOVA	ANOVA	ANOVA
ω6	p value	**0.003**	0.109	0.287
	statistics	F_3,25_ = 6.01	F_3,20_ = 2.30	F_2,15_ = 1.36
	test	ANOVA	ANOVA	ANOVA
ω3/ω6	p value	**0.026**	0.219	**0.034**
	statistics	df = 3, X^2^ = 9.26	F_3,20_ = 1.61	df = 2, X^2^ = 6.77
	test	Kruskal-Wallis	ANOVA	Kruskal-Wallis
DHA	p value	0.050	**0.019**	0.369
	statistics	df = 3, X^2^ = 7.80	F_3,20_ = 4.16	F_2,15_ = 1.07
	test	Kruskal-Wallis	ANOVA	ANOVA
LIN	p value	0.271	0.093	0.665
	statistics	df = 3, X^2^ = 3.91	df = 3, X^2^ = 6.43	F_2,15 = _ 0.419
	test	Kruskal-Wallis	Kruskal-Wallis	ANOVA
DHA/LIN	p value	**0.005**	0.119	0.291
	statistics	df = 3, X^2^ = 12.91	df = 3, X^2^ = 5.86	df = 2, X^2^ = 2.47
	test	Kruskal-Wallis	Kruskal-Wallis	Kruskal-Wallis

The statistically significant values are indicated in bold.

**Table 3:** Results from the general linear models (GLMs) applied to each stable isotope (*δ* ^15^N, *δ* ^13^C, *δ* ^34^S) and fatty acid parameter (ω3, ω6 PUFAs) with species (*Myotis myotis*, *M. daubentonii*, *M. mystacinus*) and date as explaining variables.

parameter	*M. myotis*	*M. daubentonii*	*M. mystacinus*	date
*δ* ^15^Ν	**<0.001**	**<0.001**	**<0.001**	**<0.001**
*δ* ^13^C	**<0.001**	**<0.001**	**0.033**	**<0.001**
*δ* ^34^S	**<0.001**	0.225	**0.002**	0.094
ω3 PUFAs	**<0.001**	**<0.001**	**<0.001**	0.107
ω6 PUFAs	**<0.001**	**<0.001**	0.858	**<0.001**

The statistically significant values are indicated in bold.

### Fatty acids

**Figure 4:**
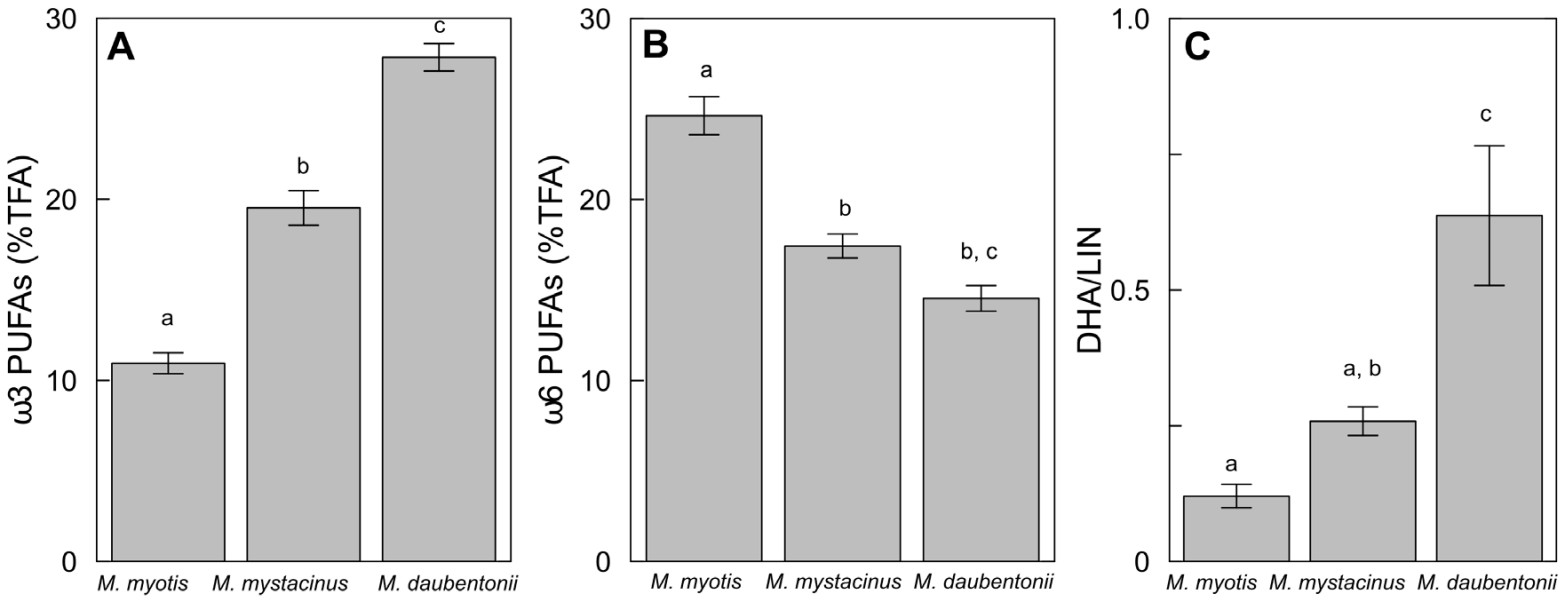
A. ω3 PUFAs, B. ω6 PUFAs and C. DHA/LIN ratio per species. Mean values ± standard errors of A. ω3 PUFAs, B. ω6 PUFAs and C. DHA/LIN ratio from the faeces of *Myotis myotis* (n = 29), *M. mystacinus* (n = 24) and *M. daubentonii* (n = 18). %TFA =  % of total fatty acid. The groups with different letter were different.

**Figure 5:**
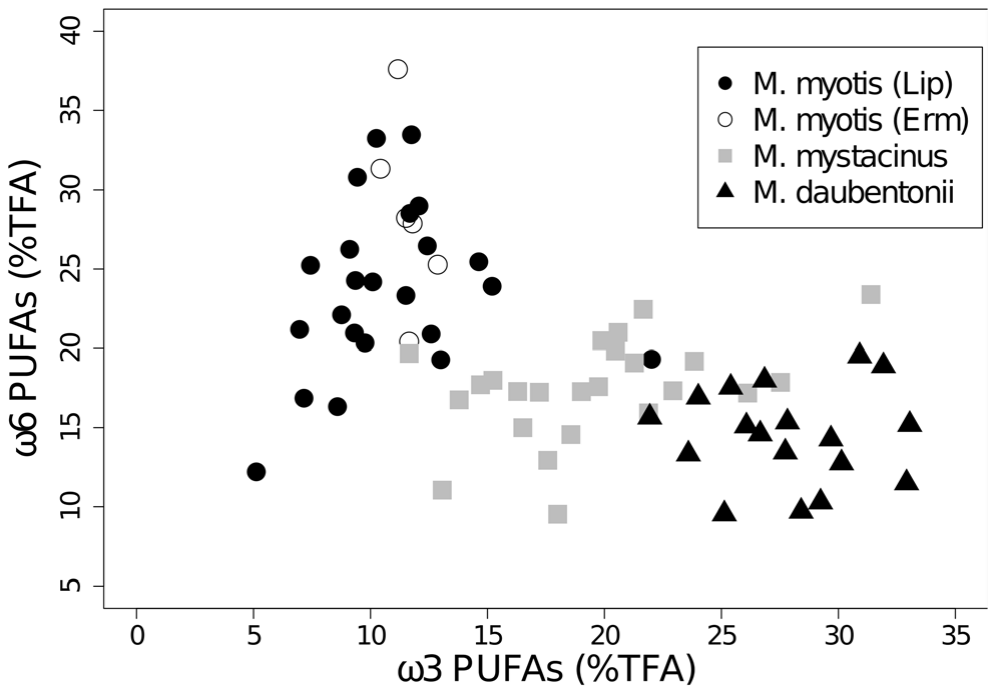
ω6 PUFAs versus ω3 PUFAs per species. Total ω6 PUFAs plotted against total ω3 PUFAs for *Myotis myotis* (n = 29), *M. mystacinus* (n = 24) and *M. daubentonii* (n = 18), TFA =  % of total fatty acid. Erm: Ermatingen, Lip: Lipperswil.

We found differences for almost all pair-wise comparisons of the concentrations of total ω3 and ω6 PUFAs, DHA, LIN, and the ratios DHA/LIN and ω3/ω6 (Table 4). Only the concentration of DHA and the ω3/ω6 ratio did not differ between *M. daubentonii* and *M. mystacinus* and the concentration of LIN was not different between *M. myotis* and *M. daubentonii* (Table 4). The faeces of *M. daubentonii* were characterized by an almost threefold higher concentration of ω3 PUFAs relative to *M. myotis* (Figure 4A, Figure 5). In contrast, the concentrations of ω6 PUFAs in the faeces of *M. myotis* were higher (by 10.28±1.27%TFA) than in *M. daubentonii* (Figure 4B, Figure 5). Both, ω3 and ω6 PUFA concentrations in the faeces of *M. mystacinus* were intermediate to the other two species (ω3: 17.43±1.85% TFA and ω6: 19.54 ±3.69% TFA) (Figure 4A, Figure 4B, Figure 5). The single PUFAs, especially DHA and to a lesser extent LIN, showed similar patterns to the total ω3 and ω6 PUFAs, respectively. DHA concentrations were higher in the faeces of *M. daubentonii* than in the faeces of *M. myotis* (mean±se: 0.31±0.05% TFA vs. 0.07±0.05% TFA, respectively) while the opposite was the case for LIN (0.54±0.04% TFA vs. 4.30±3.64% TFA, respectively). *Myotis mystacinus* had intermediate concentrations of DHA (0.22±0.03% TFA) and of LIN (0.97±0.14% TFA).

**Table 4:** Results of the ANOVAs and post-hoc tests and Kruskal-Wallis tests for the comparison of the fatty acid values between the species, using i) all the samples and ii) the samples collected in the middle of May only.

		all samples				
	ω3	ω6	ω3/ω6	DHA	LIN	DHA/LIN
p value	**<0.001**	**<0.001**	**<0.001**	**<0.001**	**0.004**	**<0.001**
statistics	df = 2, X^2^ = 54.03	df = 2, X^2^ = 37.38	df = 2, X^2^ = 57.57	df = 2, X^2^ = 29.06	df = 2, X^2^ = 10.81	df = 2, X^2^ = 31.69
species with difference	all	all	myo-dau/ myo-mys	myo-dau/ myo-mys	mys-dau/ mys-myo	all
test	Kruskal-Wallis	Kruskal-Wallis	Kruskal-Wallis	Kruskal-Wallis	Kruskal-Wallis	Kruskal-Wallis
		samples only from mid May				
	ω3	ω6	ω3/ω6	DHA	LIN	DHA/LIN
p value	**<0.001**	**<0.001**	**<0.001**	0.146	0.292	**0.013**
statistics	F_2,21_ = 49.54	F_2,21_ = 16.01	F_2,21_ = 23.43	df = 2, X^2^ = 3.85	df = 2, X^2^ = 2.46	F_2,21_ = 5.44
species with difference	all	myo-dau/ myo-mys	myo-dau	none	none	myo-dau/ mys-dau
test	ANOVA	ANOVA	ANOVA	Kruskal-Wallis	Kruskal-Wallis	ANOVA

myo: *Myotis myotis*, mys: *M. mystacinus*, dau: *M. daubentonii.* The statistically significant values are indicated in bold.

A lower ω3/ω6 ratio indicated greater consumption of terrestrial prey (*M. daubentonii*>*M. mystacinus*>*M. myotis*). The ω3/ω6 ratio was more than four times higher in *M. daubentonii* than in *M. myotis* and a similar trend occurred for the DHA/LIN ratio (Figure 4C). Interestingly, the ω3/ω6 and DHA/LIN ratios from faeces of *M. mystacinus* were relatively balanced (ω3/ω6 = 1.14±0.06 and DHA/LIN = 0.28±0.03).

Concentrations of ω6 PUFAs decreased with time in the faeces of *M.* myotis (ANOVA, F_3,25_ = 6.01, p = 0.003) and less pronouncedly in the faeces of *M. daubentonii* (ANOVA, F_2,15_ = 1.36, p = 0.287), while a temporal decrease in concentrations of ω3 PUFAs occured in the faeces of *M. mystacinus* (ANOVA, F_3,20_ = 6.26, p = 0.004) (Figure 3, Table 2). The temporal differences in DHA and LIN concentrations were not significant for any species, except for DHA in *M. mystacinus* (ANOVA, F_3,20_ = 4.16, p = 0.019) (Figure 3, Table 2). When we compared the samples collected during the same days (mid-May), total ω3, total ω6 and ω3/ω6 values were different (Tukey’s HSD, p<0.005) between all pairs of species, except ω6 PUFAs between *M. mystacinus* and *M. daubentonii* (ANOVA, F_2,21_ = 16.01, p = 0.677) and the ω3/ω6 ratio between *M. mystacinus* and the other two species (Table 4). The ratio DHA/LIN was different between the species (ANOVA, p<0.05) except for *M. mystacinus* vs. *myotis* (ANOVA, F_2,21_ = 5.44, p = 0.633). The DHA and LIN concentrations did not differ between the species (Kruskal-Wallis, p>0.146) (Table 4). The GLMs for the PUFAs indicated that the variation of ω3 PUFAs was explained mainly by species (p<0.001) with date not significant (p = 0.107), while for ω6 PUFAs, both species (except *M. mystacinus*) and date were significant (p<0.001) (Table 3).

### Linear discriminant function analysis

The linear discriminant analysis based on stable isotope values successfully assigned all faecal samples of *M. myotis* and *M. daubentonii* to the correct species (100%; Table 5). In 87% of the cases, *M. mystacinus* samples were attributed correctly. The rest were misclassified as *M. myotis* or *M. daubentonii*. The linear discriminant analysis based on fatty acids (ω3, ω6 and ω3/ω6) performed less well, yet was still able to classify faeces to the correct species, 97% of *M. myotis* faeces and 83% of *M. daubentonii* and *M. mystacinus* faeces were assigned correctly (Table 5).

**Table 5:** Results of the linear discriminant function analysis for the stable isotope (*δ* ^15^N, *δ* ^13^C, *δ* ^34^S) and fatty acid data (total ω3 and ω6 PUFAs and ω3/ω6 ratio).

	Species		Predicted classification			
	Prior classification	*M. myotis*	*M. mystacinus*	*M. daubentonii*		Prediction success
with stable isotopes	*M. myotis*	29	0	0		100.00
	*M. mystacinus*	1	20	2		86.96
	*M. daubentonii*	0	0	29		100.00
	Prior classification	*M. myotis*	*M. mystacinus*	*M. daubentonii*	Prediction success	
with fatty acids	*M. myotis*	28	1	0		96.55
	*M. mystacinus*	1	20	3		83.33
	*M. daubentonii*	0	3	15		83.33

## Discussion

Both tracers, stable isotope and fatty acid signatures, successfully discriminated among the three study species in terms of the aquatic and terrestrial origins of their diet, consistent with known feeding preferences. The fact that there was no difference in stable isotope and fatty acid signatures between the faecal samples from the lake-near and the lake-far roosts of *M. myotis* confirms that individuals of this species strictly rely on terrestrial prey irrespective of proximity to aquatic ecosystems.

### Stable isotopes

Faecal stable isotope signatures showed that the three species occupy different isotopic niches consistent with our predictions: *M. myotis* feeds on terrestrial prey and had higher *δ* ^13^C and lower *δ* ^15^N values than *M. daubentonii,* an ecological specialist who forages over water bodies where catches insects as they emerge. The indication of an aquatic, a terrestrial or a mixed diet signature was more distinct based on *δ* ^15^N than on *δ* ^13^C or *δ* ^34^S values. The more a species depended on aquatic food, the higher *δ* ^15^N values (*δ* ^15^N: *M. myotis* < *M. mystacinus* < *M. daubentonii*). It is also possible that higher *δ* ^15^N values imply feeding on prey from higher trophic levels. Although we cannot exclude piscivory by *M. daubentonii* as it has been shown that it is able to catch small fish [37], this is rather unlikely since there are no studies reporting fish remnants in the faeces of this species.

The intermediate values of *δ* ^15^N, the concentrations of ω3 and ω6 PUFAs, the concentration of DHA and the ω3/ω6 and DHA/LIN ratios recorded in the faeces of *M. mystacinus* were indicative of the mixed diet of this species. This would not have been revealed as clearly by using a single stable isotope (*δ* ^13^C, *δ* ^34^S). The triple approach (*δ* ^13^C, *δ* ^15^N and deuterium) was also successful in identifying breeding origins of migrating bats [38]. The higher variability in the isotope signatures in the values of *M. daubentonii* and *M. mystacinus* compared to *M. myotis* (Figure 2) is likely due to a higher variability in the diet or/and in the feeding habitat [39]. However, since the species are feeding in different systems a direct comparison of diet breadth was not possible as the dietary baseline signatures are different.

Our study includes the calculation of *δ* ^34^S signatures, rarely used in mammalian ecology. Signatures of *δ* ^34^S can provide paleo-dietary information for mammals [40], detect sulphur polluted diets [41] or be applied when the species feed near the sea or prey from water with different salinities, as the *δ* ^34^S signature is related to salinity [42]. Since *δ* ^34^S can refine information obtained by *δ* ^15^N and *δ* ^13^C, our data support the recommendation that it should be routinely used [43]. Our results suggest a difference in *δ* ^34^S values between systems, with higher *δ* ^34^S for bats that rely on aquatic prey than on terrestrial, which provides additional evidence for differences in aquatic versus terrestrial organic material.

### Fatty acids

The faecal PUFA profiles, similar to our stable isotope data, indicate that *M. myotis* and *M. daubentonii* occupy different niches, while *M. mystacinus* had some overlap, in terms of aquatic and terrestrial origin of prey (Figure 5). The PUFA profiles reflected, as expected, feeding preferences for terrestrial and/or aquatic prey. Faeces of *M. myotis* had higher concentrations of linoleic acid (LIN) and total ω6 PUFAs than *M. daubentonii*, in line with our expectations. Faeces of *M. daubentonii*, known to eat aquatic prey, had significantly higher concentrations of ω3 PUFAs. The trends for higher ω3 PUFAs indicating a more aquatic diet and higher ω6 PUFAs indicating a more terrestrial diet agree with other studies on terrestrial and aquatic animals [23], [24], [44].

The ω3/ω6 and DHA/LIN ratios decreased with increasing terrestrial prey the species is assumed to consume. Similar tendencies have been reported for stream food webs (macroinvertebrates, allochthonous and autochthonous matter) [45] and semi-aquatic mammals [24]. While *M. myotis* had low ω3/ω6 and DHA/LIN ratios due to its terrestrial diet, *M. daubentonii* had the highest ratios, and *M. mystacinus* had intermediate values in ω3/ω6 and DHA/LIN ratios and thus obviously relied on a mixed diet. Presumably, *M. mystacinus* consumed more aquatic than terrestrial prey, as its PUFA profile was closer to that of *M. daubentonii.* This proximity was also evident in the stable isotope values.

### Temporal variation

The temporal variation in the tracers may be related to temporal differences in prey availability. During the study period the average air temperature gradually increased (from 14.7°C to 24.7°C) likely resulting in changes in relative abundance of certain prey species or the general composition of the insect community the bats fed on (e.g. [46], [47]). Also the stable isotope and fatty acid signatures of the insects may have changed seasonally (e.g. [48], [45]). We followed a non-invasive approach, so did not catch bats to obtain faeces from individuals of known age or sex. This might have contributed to the differences in diet as for example the energy demand of female bats is increased during pregnancy and lactation period [49] and our samples probably included faeces from bats in different reproductive stages. Differences in hair stable isotopes (*δ* ^13^C, *δ* ^15^N, *δ* ^34^S, hydrogen) do occur between males and females, and juvenile and adult individuals of the insectivorous bat, *Eptesicus fuscus* [50].

Almost all parameters (*δ* ^13^C, *δ* ^15^N, *δ* ^34^S, ω3 and ω6 PUFAs) differed between the three species, no matter whether we compared only the samples collected in the same period or all the samples. This implies that the stable isotope and fatty acid signatures of aquatic and terrestrial prey, regardless of the temporal variation we found, are different. It also indicates that these bats have stable preferences for aquatic or terrestrial prey, consistent with the conclusions from studies employing identification of prey remains in faeces.

### Stable isotope vs. fatty acid analysis. Applications in ecology

Our results indicate a complementarity between the two tracers. The linear discriminant analysis assigned faeces to the correct species with similar success for both stable isotope and fatty acid signatures. Although absolute stable isotope values can differ between regions, dissimilarities also occur between different habitats within one region. The spatial scale at which stable isotope analyses remain comparable may differ, depending on the heterogeneity of the habitat. Thus, caution must be taken when comparing isotopic values of different regions and the baseline isotopic signatures must be known [51].

While stable isotope signatures depend on the region, differences in fatty acids between aquatic and terrestrial systems are more universal. Thus, when baseline isotopic signatures are unknown for the study area, fatty acid signatures may be the preferred tracers. Stable isotopes, however, are superior when information for the individual level is required or when there is low availability of samples, as stable isotope analysis can be conducted even on a single faecal pellet. Fatty acid analysis, typically requires larger quantities of sample, which means that one sample has to be comprised of more than one pellet. If these are collected from a roost, fatty acid signatures reflect an integrated signature.

Since faeces [52] and their stable isotopes [25] both provide information about the most recent food, they can be used to track short-term diet or habitat shifts and flexibility in feeding behaviour. Isotope signatures do not allow identification of prey items to the species level, as molecular or taxonomic analysis of the fragments would do, but they do allow tracking the type of feeding habitats. Investigating the aquatic versus terrestrial feeding preferences of the species could be used, for instance, for the conservation of endangered species, especially in areas with limited water resources. By examining the contribution of aquatic food in the diet of terrestrial predators, it is possible to determine which species depend on aquatic prey and predict possible consequences for these species in case of aquatic habitats eliminations and degradations.

Further questions that need to be addressed include the potential importance of certain fatty acids originating from different habitats in determining food quality for mammals and whether or not the availability of these fatty acids is associated with fitness consequences. In mammals, dietary deficiencies in ω3 PUFAs have been related to behavioural disorders [53].

In conclusion, we show that fatty acids (i.e., total ω3 PUFAs, ω6 PUFAs or single fatty acids such as DHA and LIN or their ratios) and stable isotopes (i.e., *δ* ^13^C, *δ* ^15^N, *δ* ^34^S) in faeces can be used as ecological tracers for aquatic and terrestrial diet preferences of bats and potentially other mammals. Faeces can be used as an alternative to animal tissue when investigating recent diet and represent a non-invasive approach.
